# Isolation of the bioactive peptides CCHamide-1 and CCHamide-2 from *Drosophila* and their putative role in appetite regulation as ligands for G protein-coupled receptors

**DOI:** 10.3389/fendo.2012.00177

**Published:** 2012-12-31

**Authors:** Takanori Ida, Tomoko Takahashi, Hatsumi Tominaga, Takahiro Sato, Hiroko Sano, Kazuhiko Kume, Mamiko Ozaki, Tetsutaro Hiraguchi, Hajime Shiotani, Saki Terajima, Yuki Nakamura, Kenji Mori, Morikatsu Yoshida, Johji Kato, Noboru Murakami, Mikiya Miyazato, Kenji Kangawa, Masayasu Kojima

**Affiliations:** ^1^Interdisciplinary Research Organization, University of MiyazakiMiyazaki, Japan; ^2^Department of Biology, School of Medicine, Kurume UniversityFukuoka, Japan; ^3^Molecular Genetics, Institute of Life Sciences, Kurume UniversityFukuoka, Japan; ^4^Department of Stem Cell Biology, Institute of Molecular Embryology and Genetics, Kumamoto UniversityKumamoto, Japan; ^5^Department of Biology, Graduate School of Science, Kobe UniversityHyogo, Japan; ^6^Department of Biochemistry, National Cerebral and Cardiovascular Center Research InstituteOsaka, Japan; ^7^ Frontier Science Research Center, University of MiyazakiMiyazaki, Japan; ^8^Department of Veterinary Physiology, Faculty of Agriculture, University of MiyazakiMiyazaki, Japan

**Keywords:** GPCR, novel bioactive peptide, *Drosophila*, CCHamide, bombesin receptor subtype 3

## Abstract

There are many orphan G protein-coupled receptors (GPCRs) for which ligands have not yet been identified. One such GPCR is the bombesin receptor subtype 3 (BRS-3). BRS-3 plays a role in the onset of diabetes and obesity. GPCRs in invertebrates are similar to those in vertebrates. Two *Drosophila* GPCRs (CG30106 and CG14593) belong to the BRS-3 phylogenetic subgroup. Here, we succeeded to biochemically purify the endogenous ligands of *Drosophila* CG30106 and CG14593 from whole *Drosophila* homogenates using functional assays with the reverse pharmacological technique, and identified their primary amino acid sequences. The purified ligands had been termed CCHamide-1 and CCHamide-2, although structurally identical to the peptides recently predicted from the genomic sequence searching. In addition, our biochemical characterization demonstrated two N-terminal extended forms of CCHamide-2. When administered to blowflies, CCHamide-2 increased their feeding motivation. Our results demonstrated these peptides actually present as the major components to activate these receptors in living *Drosophila*. Studies on the effects of CCHamides will facilitate the search for BRS-3 ligands.

## INTRODUCTION

G protein-coupled receptors (GPCRs) constitute a large protein superfamily that shares a 7-transmembrane motif as a common structure. Human genome sequencing has identified several hundred orphan GPCRs for which ligands have not yet been identified (Vassilatis et al., 2003). GPCRs play crucial roles in cell-to-cell communication involved in a variety of physiological phenomena and are the most common target of pharmaceutical drugs. Therefore, the identification of endogenous ligands for orphan GPCRs will lead to clarification of novel physiological regulatory mechanisms and potentially facilitate the development of new GPCR-targeted therapeutics. Many bioactive molecules have been discovered or identified as endogenous ligands of orphan GPCRs through reverse pharmacology to date (Civelli et al., 2012). These molecules include nociceptin, prolactin-releasing peptide, orexin, apelin, ghrelin, metastin, and neuromedin S. The discovery of novel endogenous ligands for orphan GPCRs in mammals is currently challenging, possibly because of the restricted timing of expression or distribution of GPCR ligands. One orphan receptor in mammals is the bombesin receptor subtype 3 (BRS-3). BRS-3 is primarily expressed in the hypothalamus and plays a role in the onset of diabetes and obesity (Ohki-Hamazaki et al., 1997). Although several small molecules that are agonists and antagonists for BRS-3 have been synthesized, the native ligand of BRS-3 has not yet been identified (Majumdar and Weber, 2012).

The recent sequencing of the *Drosophila melanogaster* genome has enabled the identification of at least 160 fly GPCRs (Brody and Cravchik, 2000). *Drosophila* is an excellent animal model for genetic analysis of developmental and behavioral processes, as it is a small, genetically modifiable organism with a relatively short lifecycle and can be bred easily under laboratory conditions. Structural or sequence comparison of newly discovered peptides in *Drosophila* with candidate molecules in mammals may lead to the discovery of new peptide signaling modules. We recently reported the discovery of dRYamide-1, dRYamide-2, and trissin as ligands for *Drosophila* orphan GPCRs (Ida et al., 2011a,b). We consider it likely that additional novel bioactive peptides can be discovered for orphan GPCRs. Two *Drosophila* GPCRs (CG14593 and CG30106) belong to the BRS-3 phylogenetic subgroup (Hewes and Taghert, 2001).

Here, we report the identification of CCHamide-1 and CCHamide-2, which are ligands for GPCRs CG30106 and CG14593, respectively, in *D. melanogaster*. Injection of CCHamide-2 resulted in the stimulation of feeding motivation in blowflies. These bioactive peptides may provide new insights in the search for BRS-3 ligands and the elucidation of *D. melanogaster* feeding mechanisms.

## MATERIALS AND METHODS

### PURIFICATION OF *Drosophila* CCHamide-1 AND CCHamide-2

An assay system using CG30106- or CG14593-expressing cells was prepared as previously described (Ida et al., 2011a,b). The full-length cDNA of *Drosophila* CG30106 (GenBank accession number: NM_136355; residues -31 to 1700) and CG14593 (GenBank accession number: NM_136355; residues 656–2185) was obtained by RT-PCR using *Drosophila* cDNA as the template. The sense and antisense primers for CG30106 were 5′-aaatcgagcggactcagtacat-3′ and 5′-gtggcctgtaattcctgtaaactc-3′, respectively. The sense and antisense primers for CG14593 were 5′-tgagacatcttgcccaggag-3′ and 5′-gtgtttcggtacctccatttat-3′, respectively. The amplified cDNA was ligated into the pcDNA3.1 vector (Invitrogen). The expression vector, i.e., CG30106 or CG14593-pcDNA3.1, was transfected into Chinese hamster ovary (CHO) cells by using with Fugene6 transfection reagent (Roche), and stably expressing cells were selected using 1 mg/ml G418. The selected cell line, i.e., CHO-CG30106-line 2-4 or CHO-CG14593-line 10-1, showed the highest expression of CG3106 or CG14593 mRNA, respectively. Cells were cultured in a humidified environment of 95% air and 5% CO_2_. Changes in intracellular Ca^2^^+^ concentrations ([Ca^2^^+^]_i_) were measured using the FlexStation 3 fluorometric imaging plate reader to conduct high-throughput measurements of intracellular Ca^2^^+^ concentration (Molecular Devices, CA, USA; Marshall et al., 2005). CHO-CG30106 or CHO-CG14593 cells (3 × 10^4^ cells) were plated into 96-well black-wall microplates (Corning, NY, USA) 20 h before each assay. The cells were incubated with 100 μl of Calcium 4 assay kit reagent (Molecular Devices) for 1 h, and then 50 μl of each sample was added to the CHO-CG30106 or CHO-CG14593 cells to induce changes in fluorescence. The maximum [Ca^2^^+^]_i_ changes were recorded.

*Drosophila melanogaster* flies (Canton S.; 350 g) were collected on dry ice. The whole body of each fly was boiled for 10 min in 10 volumes of water to inactivate intrinsic proteases. The solution was adjusted to 1 M AcOH. Peptides were extracted by homogenization using a Polytron mixer. The supernatant of the extracts, obtained after 30 min of centrifugation at 11,000 rpm, was concentrated to approximately 1/10 by an evaporator. The residual concentrate was subjected to acetone precipitation using 66% acetone. After the precipitates were removed, the supernatant acetone was evaporated and loaded onto a 40-g cartridge of Sep-Pak C18 (Waters), which was pre-equilibrated with 0.1% trifluoroacetic acid (TFA). The Sep-Pak cartridge was washed with 10% CH_3_CN/0.1% TFA, and then eluted with 60% CH_3_CN/0.1% TFA. The eluate was evaporated and lyophilized. The residual materials were redissolved in 1 M AcOH and then adsorbed on a column of SP-Sephadex C-25 (H^+^ form) that had been pre-equilibrated with 1 M AcOH. Successive elutions with 1 M AcOH, 2 M pyridine, and 2 M pyridine–AcOH (pH 5.0) provided three fractions of SP-I, SP-II, and SP-III. A basic peptide fraction (SP-III) was fractionated on a Sephadex G-50 gel filtration column (2.9 cm × 142 cm; GE Healthcare, Tokyo, Japan). A portion of each fraction, equivalent to 1.16 g of flies, was subjected to the assay using CHO-CG30106 or CHO-CG14593 cells. The active fraction was separated by carboxymethyl (CM)-ion-exchange high-performance liquid chromatography (HPLC) on a TSK CM-2SW column (4.6 mm × 250 mm; Tosoh, Tokyo, Japan) with an ammonium formate (HCOONH_4_; pH 6.5) gradient of 10 mM to 1 M in the presence of 10% acetonitrile (ACN) at a flow rate of 1 ml/min for 160 min. The active fractions were separated by reverse-phase (RP)-HPLC with a μBondasphere C18 column (3.9 mm × 150 mm, Waters, MA, USA) by using a 10–60% ACN/0.1% TFA linear gradient at a flow rate of 1 ml/min for 80 min. The active fractions were further purified by RP-HPLC using a diphenyl column (2.1 mm × 150 mm, 219TP5125; Vydac, Hesperia, CA, USA) for 80 min by using a linear gradient of 10–60% ACN/0.1% TFA at a flow rate of 0.2 ml/min. Fractions corresponding to absorption peaks were collected, and an aliquot of each fraction (2 g tissue equivalent) was assayed by using the FLEX system. The active fractions were further purified by RP-HPLC by using a Chemcosorb 3ODSH column (2.1 mm × 75 mm; Chemco, Osaka, Japan) for 80 or 160 min by using a linear gradient of 10–60% ACN/0.1% TFA at a flow rate of 0.2 ml/min. Fractions corresponding to absorption peaks were collected, and an aliquot of each fraction (2 g tissue equivalent) was assayed by using the FLEX system. Approximately 20 pmol of the final purified peptides was analyzed using a protein sequencer (model 494; Applied Biosystems, CA, USA), and approximately 1 pmol of each active fraction was subjected to determination of molecular weight by matrix-assisted laser desorption–ionization time of flight (MALDI-TOF) mass spectrometry by using a Voyager-DE PRO instrument (Applied Biosystems).

### CLONING OF *Drosophila* PREPRO-CCHamide-1 AND CCHamide-2 cDNA

A tBLASTn search of the *Drosophila* genome resources was performed by using sequence of the purified peptides, and we obtained *D. melanogaster* mRNA sequences [CG14358 (CCHamide-1), NM_001104314; and CG14375 (CCHamide-2), NM_142028] derived from an annotated genomic sequence. We searched for open reading frames upstream and downstream of the genome sequences of CCHamide-1 and CCHamide-2 by using specific primers 5′-cgtgcagcttgcgaaataata-3′ and 5′-cttctggcttagctagcgtgttatc-3′ for CCHamide-1 and 5′-caccagccaagtgcaagtatc-3′ and 5′-cggtttttaatgtacgttgtgg-3′ for CCHamide-2. The candidate PCR product was subcloned into the pCR-II TOPO vector and sequenced. The nucleotide sequence of the isolated cDNA fragment was determined by automated sequencing (DNA sequencer model 3100; Applied Biosystems) according to the protocol for the BigDye terminator cycle sequencing kit (Applied Biosystems).

### PEPTIDES

CCHamide-1 (SCLEYGHSCWGAH-NH_2_), CCHamide-2 (GCQAYGHVCYGGH-NH_2_), CCHamide-1 C-terminal free (SCLEYGHSCWGAH), CCHamide-2 C-terminal free (GCQAYGHVCYGGH), long-form CCHamide-2 (AQQSQAKKGCQAYGHVCYGGH-NH_2_), and long-form CCHamide-2 C-terminal free (AQQSQAKKGCQAYGHVCYGGH) were synthesized by Peptide Institute Inc. (Osaka, Japan).

### PROBOSCIS EXTENSION REFLEX TEST FOR APPETITE MEASUREMENT

The proboscis extension reflex (PER) test and feeding test were performed for the blowfly* Phormia regina* as previously described (Nisimura et al., 2005; Ida et al., 2011a). CCHamide-2 was dissolved in blowfly linger solution at a concentration of 10 pμol/ml. Twenty flies were secured by their wings using washing pins, and the first PER test was performed by using 12 steps of sucrose concentrations that had been prepared by twofold serial dilutions in distilled water, beginning from a sucrose concentration of 1 M. We investigated the PER in three different groups of 20 flies each: no injection, fly linger injection, and fly linger plus peptide injection. The PER tests were performed 30 min after 1 μl of blowfly linger solution with or without peptide was injected into the shoulder of each fly. We repeated five sets of PER tests each, in which 20 flies were used in each batch.

### STATISTICAL ANALYSIS

Results are presented as the mean ± SEM for each group. To compare the PER thresholds among the three groups, we used a non-parametric Steel–Dwass test. The criterion for statistical significance was *p* < 0.05 for all tests. The statistical software program GraphPad PRISM (GraphPad software, CA, USA) was used for analyses.

## RESULTS

### STRUCTURAL DETERMINATION OF CCHamide-1 FOR CG30106

**FIGURE 1:**
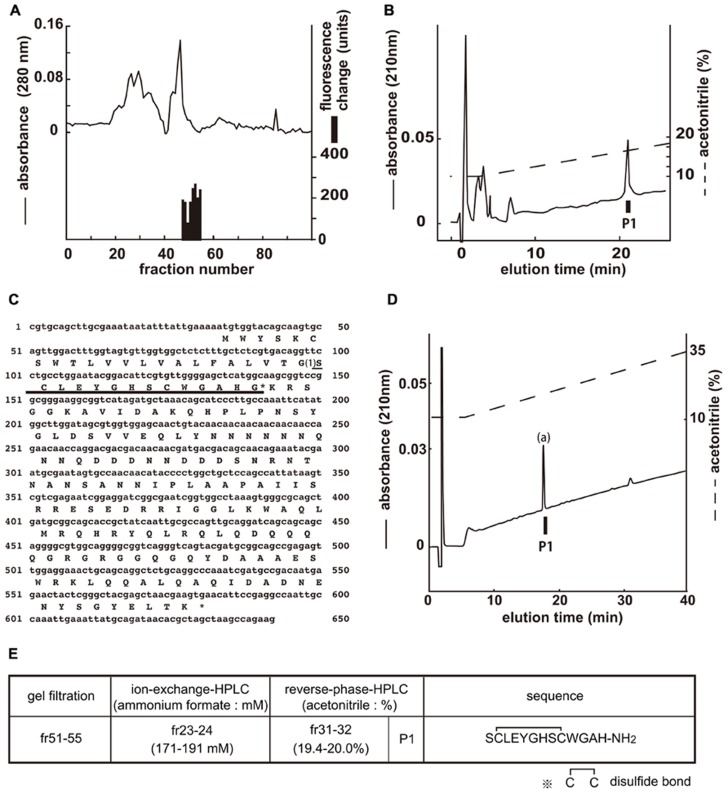
**Purification of CCHamide-1 from fly extracts**. *Black bars* indicate changes of [Ca^2^^+^]_i_ fluorescence signal in CHO-CG30106 cells. **(A)** G-50 gel filtration of the SP-III fraction of fly extracts. The active fraction was subjected to one step of CM-ion-exchange HPLC and three steps of RP-HPLC. **(B)** Final purification of the active fraction by RP-HPLC. **(C)** Nucleotide sequence and deduced amino acid sequence of CCHamide-1 cDNA. CCHamide-1 cDNA encode 182-residue peptides. The asterisk indicates a glycine residue that serves as an amide donor for C-terminal amidation. The CCHamide-1 sequence is underlined as (1). **(D)** Chromatographic comparison by RP-HPLC of natural CCHamide-1 and synthetic CCHamide-1. *Black bar* (P1) indicates the changes of [Ca^2^^+^]_i_ fluorescence signal in CHO-CG30106 cells. Each peptide was applied to a Symmetry C18 column (3.9 mm × 150 mm, Waters, MA, USA) with a 10–60% ACN/0.1% trifluoroacetic acid (TFA) linear gradient at a flow rate of 1 ml/min for 80 min. P1 represent active fraction containing natural CCHamide-1. (a) Synthetic CCHamide-1. **(E)** Active fractions of each chromatography and the amino acid sequence of CCHamide-1.

[Ca^2^^+^]_i_ assays were performed by using the gel filtration samples to isolate the endogenous ligands of CG30106 (**Figure 1A**). The active fractions were observed in eight sequential fractions (numbers 48–55). The fractions (51–55) with particularly high activity were separated by CM-ion-exchange HPLC at pH 6.5. The active fractions were separated by RP-HPLC. The active fraction was purified as a single peak in the final RP-HPLC (**Figure 1B**, P1). The amino acid sequence of the purified peptide was determined as SXLEYGHSXWGAH (P1; where X is a position that was not identified) using a protein sequencer. To elucidate the complete amino acid sequence of this peptide, *Drosophila* cDNA encoding the purified peptides was isolated by RT-PCR. The cDNA encoded a 182-residue protein (CG14358; **Figure 1C**) that contained features characteristic of an N-terminal signal peptide immediately preceding the purified peptide sequence. Every X residue was a cysteine, and the rest of the sequence was identical to that determined by peptide sequencing (**Figure 1C**). Sequencing resulted in a very low yield of phenyl thiohydantoin (PTH) at the steps involving X, which suggests that two cysteines may form disulfide bonds (S–S bonds). The preproprotein contained a potential processing site at the C-terminal end of the purified peptide sequence. This peptide contained Gly residues that presumably serve as an amide donor for C-terminal amidation. We therefore deduced the primary structure of the peptide to be SCLEYGHSCWGAH-NH_2_. This peptide had been named CCHamide-1 (Roller et al., 2008). Mass spectrometric analysis revealed that the observed monoisotopic *m/z* value of the purified peptide (1445.30) was very similar to the theoretically predicted value for this peptide (1445.55) when including an intrachain disulfide bond and C-terminal amidation. We generated the synthetic peptide SCLEYGHSCWGAH-NH_2_ (CCHamide-1). The retention time of the P1 active fraction was identical to that of the synthetic SCLEYGHSCWGAH-NH_2_ peptide (which has an intrachain disulfide bond) on RP-HPLC (**Figure 1D**). Thus, these data suggest that both natural peptides have an intrachain disulfide bond and C-terminal amidation. **Figure 1E** shows the active fractions of each chromatography and the amino acid sequence of CCHamide-1.

### STRUCTURAL DETERMINATION OF CCHamide-2 FOR CG14593

**FIGURE 2:**
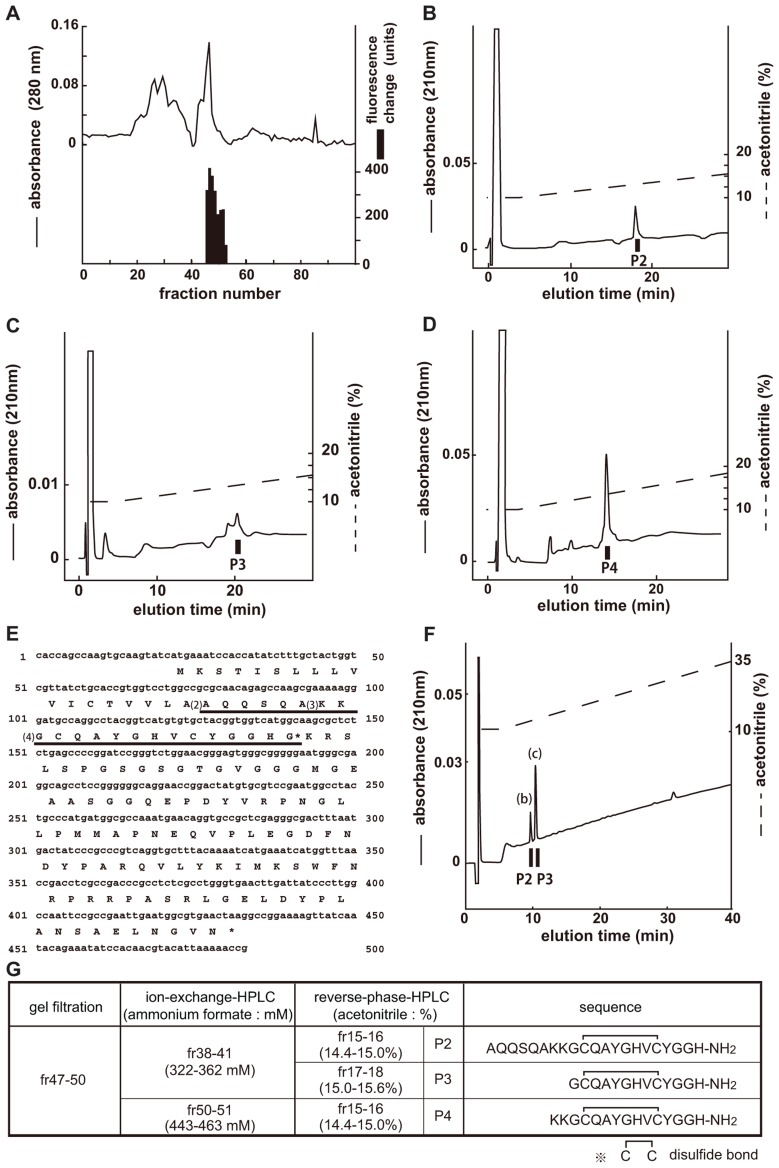
**Purification of CCHamide-2 from fly extracts**. *Black bars* indicate changes of [Ca^2^^+^]_i_ fluorescent signal in CHO-CG14593 cells. **(A)** G-50 gel filtration of the SP-III fraction of fly extracts. The active fraction was subjected to one step of CM-ion-exchange HPLC and three steps of RP-HPLC. **(B–D)** Final purification of the active fraction by RP-HPLC. **(E)** Nucleotide sequence and deduced amino acid sequence of CCHamide-2 cDNA. CCHamide-2 cDNA encodes a 136-residue peptides. The asterisk indicates a glycine residue that serves as an amide donor for C-terminal amidation. The CCHamide-2 sequence is underlined as (4). The other long-form of CCHamide-2 is translated from (2) or (3). **(F)** Chromatographic comparison by RP-HPLC of natural CCHamide-2 and synthetic CCHamide-2. *Black bars* (P2, P3) indicate the changes of [Ca^2^^+^]_i_ fluorescence signal in CHO-CG14593 cells. Each peptide was applied to a Symmetry C18 column with a linear gradient elution for 80 min. P2 and P3 represent active fractions containing natural CCHamide-2. (b) Synthetic long-form of CCHamide-2. (c) Synthetic CCHamide-2. **(G)** Active fractions of each chromatography and the amino acid sequence of CCHamide-2.

The endogenous ligands of CG14593 were isolated in the same manner as those of CG30106 (**Figure 2A**). Three separate active fractions were revealed (**Figure 2G**, P2, P3, and P4), and each active fraction was purified as a single peak in the final RP-HPLC (Figures 2B–D). From the results obtained by using a protein sequencer and *Drosophila* cDNA encoding the purified peptides (**Figure 2E**), we deduced the primary structure of the peptide to be AQQSQAKKGCQAYGHVCYGGH-NH_2_ (P2), GCQAYGHVCYGGH-NH_2_ (P3), and KKGCQAYGHVCYGGH-NH_2_ (P4; **Figure 2G**). All of these cysteines may form S–S bonds. The shortest peptide (P3) had been named CCHamide-2. The cDNA encoded a 136-residue protein (CG14375; **Figure 2E**) that contained features characteristic of an N-terminal signal peptide immediately preceding the purified longest peptide sequence (P2). All peptides were derived from the same precursor (CG14375), but the length of the N-terminal peptide was different. Mass spectrometric analysis revealed that the observed monoisotopic *m/z* values of the purified peptides (P2, 2216.80; P3, 1347.69; and P4, 1603.60) were similar to the theoretically predicted values (2216.99, 1347.52, and 1603.71, respectively) for a peptide that has an intrachain S–S bonds and C-terminal amidation. We generated the synthetic peptides AQQSQAKKGCQAYGHVCYGGH-NH_2_ (long-form CCHamide-2) and GCQAYGHVCYGGH-NH_2_ (CCHamide-2). The retention times of the P2 and P3 active fractions were identical to those of the synthetic AQQSQAKKGCQAYGHVCYGGH-NH_2_ and GCQAYGHVCYGGH-NH_2_ peptides (which have an intrachain disulfide bond) on RP-HPLC, respectively (**Figure 2F**). Thus, these data suggest that both natural peptides have an intrachain disulfide bond and C-terminal amidation.

### PHARMACOLOGICAL CHARACTERIZATION

**FIGURE 3:**
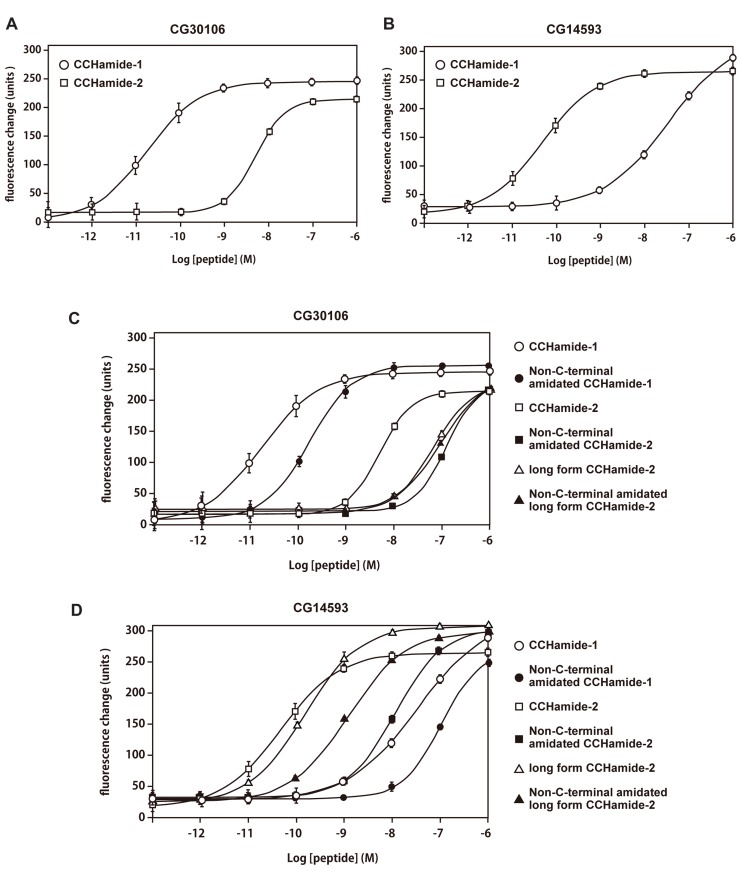
**Pharmacological characterization of synthetic peptides using CG30106 or CG14593 stably expressed in CHO cells**. **(A,B)** Concentration–response relationships of changes in [Ca^2^^+^]_i_ for CCHamide-1 (open circle) and CCHamide-2 (open square), in CHO-CG30106 cells **(A)** or CHO-CG14593 cells **(B)**. **(C,D)** Concentration–response relationships of changes in [Ca^2^^+^]_i_ for various peptides, CCHamide-1 (open circle), and CCHamide-2 (open square) in CHO-CG30106 cells **(C)** or CHO-CG14593 cells **(D)**. Non-C-terminal amidated CCHamide-1 (filled circle), non-C-terminal amidated CCHamide-2 (filled square), long-form CCHamide-2 (open triangle), and non-C-terminal amidated long-form CCHamide-2 (filled triangle). Each symbol on the line graph represents the mean ± SEM of data from six replicates for each experiment.

The interaction of CCHamide-1 and CCHamide-2 with CG30106 or CG14593 was examined using synthetic peptides. CCHamide-1 induced concentration-dependent, robust increases in [Ca^2^^+^]_i_ in CHO-CG30106 cells, with a half-maximal response concentration (EC_50_) of 1.80 × 10^-^^11^ M (**Figure 3A**). CCHamide-2 potently activated CG30106 (EC_50_; 4.86 × 10^-^^9^ M (**Figure 3A**). CCHamide-2 induced dose-dependent, robust increases in [Ca^2^^+^]_i_ in CHO-CG14593 cells, with an EC_50_ of 4.80 × 10^-^^11^ M (**Figure 3B**). CCHamide-1 potently activated CG14593 (EC_50_; 3.32 × 10^-^^8^ M (**Figure 3B**). Neither CCHamide-2 nor CCHamide-1 induced a response in CHO cells transfected with the vector alone (data not shown). In the investigation of the interaction between non-C-terminal amidated synthetic peptides or long-form CCHamide-2 and CG30106, the EC_50_ values were as follows: non-C-terminal amidated CCHamide-1, 1.66 × 10^-^^10^ M; non-C-terminal amidated long-form CCHamide-2, 8.93 × 10^-^^8^ M; long-form CCHamide-2, 6.45 × 10^-^^8^ M; and non-C-terminal amidated CCHamide-2, 1.22 × 10^-^^7^ M (**Figure 3C**). For CG14593, the EC_50_ values were as follows: long-form CCHamide-2, 1.49 × 10^-^^10^ M; non-C-terminal amidated long-form CCHamide-2, 1.18 × 10^-^^9^ M; non-C-terminal amidated CCHamide-2, 1.13 × 10^-^^8^ M; and non-C-terminal amidated CCHamide-1, 1.02 × 10^-^^7^ M (**Figure 3D**).

### PER TEST FOR MEASURING FEEDING SENSITIVITY

**FIGURE 4:**
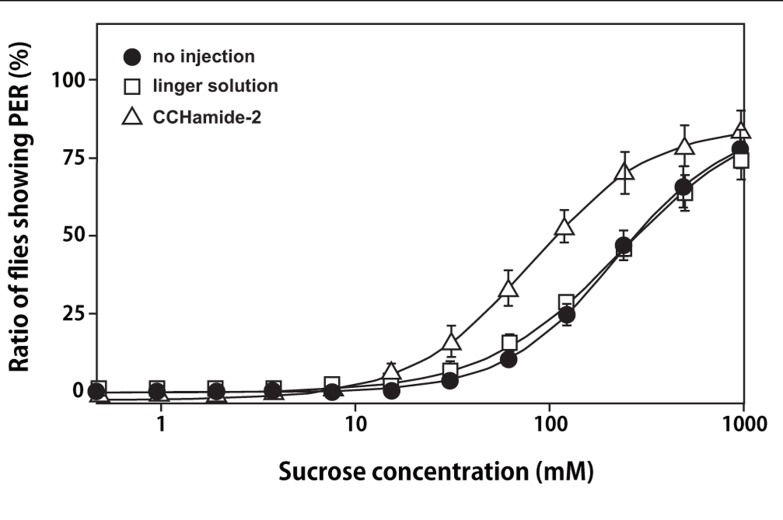
**Effect of CCHamide-2 on PER of the blowfly**. Sigmoidal curves show the sucrose concentration–PER relationship for three fly groups: no injection (closed circle), injection with linger solution (open square), and injection of CCHamide-2 (open triangle). Each symbol on the line graph represents the mean ± SEM of data from five replicates for each experiment.

As shown in **Figure 4**, a significant decrease was observed in the mean PER threshold, which was defined as the sucrose concentration at which 50% of flies show PER, after the injection of 10 pmol of CCHamide-2: the mean PER threshold decreased from 236 mM (30 min after linger solution injection) to 77.2 mM (30 min after CCHamide-2 injection; *p* < 0.05). In contrast, no difference was observed between the mean PER threshold without any injection and 30 min after linger solution injection (222 and 236 mM, respectively, *p* > 0.05).

## DISCUSSION

In this study, we biochemically purified 2 *Drosophila* peptides (CCHamide-1 and CCHamide-2) as endogenous ligands for *Drosophila* GPCRs CG30106 and CG14593. Recently, Hansen et al. (2011) independently identified these peptides from genome database and reported that synthetic CCHamide-1 and CCHamide-2 potently activated CHO/G-16 cells expressing recombinant CG30106 and CG14593. Then, Reiher et al. (2011) characterized CCHamide-1 and CCHamide-2 from the *Drosophila* midgut by capillary offline RP-HPLC coupled with MALDI-TOF MS/MS. Our biochemical characterization, however, for the first time, demonstrated three forms of CCHamide-2. The CCHamide-2 preproprotein is 136 amino acid residues long and contains three forms of CCHamide-2. The CCHamide-1 preproprotein is 182 amino acid residues long and contains one form of CCHamide-1. Pharmacological characterization by using CHO cells expressing GPCRs indicated that CCHamide-1 had a high potency for activating recombinant CG30106, but CCHamide-2 rather potently activated CG30106. In contrast, CCHamide-2 had a high potency for activating recombinant CG14593, but CCHamide-1 rather potently activated CG14593. Long-form CCHamide-2 and CCHamide-2 shared a highly similar potency for activating recombinant CG14593. Although we did not generate synthetic KKGCQAYGHVCYGGH-NH_2_, it is predicted to have a high potency similar to that of other forms of CCHamide-2 for activating CG14593 because of the relationship between the amount of purified peptide and the specific activity. KKGCQAYGHVCYGGH-NH_2_ (P4) and AQQSQAKKGCQAYGHVCYGGH-NH_2_ (P2) may be incomplete processing intermediates of GCQAYGHVCYGGH-NH_2_ (P3), originating from two alternative signal peptide cleavage sites and incomplete KK prohormone convertase processing. The quantity of the purified peptide could not be accurately measured at the time of the experiments. Because the gel filtration fractions with particularly high activity were separated by CM-ion-exchange HPLC at pH 6.5, we did not purify all peptides for their receptors from the flies collected. However, we purified peptide KKGCQAYGHVCYGGH-NH_2_ (P4) > AQQSQAKKGCQAYGHVCYGGH-NH_2_ (P2) > GCQAYGHVCYGGH-NH_2_ (P3) in amount. Therefore, in this study, we cannot conclude whether P4 and P2 are mature peptides or incomplete processing intermediates of P3. Because both CCHamide-1 and CCHamide-2 have a disulfide bond and a YGH motif, the disulfide bond is predicted to be an important structure for GPCR activation. Additionally, both peptides have a GXG-NH_2_ motif at the C-terminus. Therefore, we synthesized non-C-terminal amidated peptides to determine whether the C-terminal amide was necessary for the activation of each receptor. These results show that these peptides are considered to require both disulfide bonds and C-terminal amides to activate their respective GPCRs. Because we biochemically purified these ligands for the receptors by using the reverse pharmacological technique, we propose that no further modified forms or unknown ligands exist for these receptors in the fruit fly. CCHamide-1 is a cognate ligand for CG30106 and the three forms of CCHamide-2 are cognate ligands for CG14593.

BRS-3 is a mammalian orphan receptor (Ohki-Hamazaki et al., 1997). *Drosophila* CG30106 and CG14593 belong to the BRS-3 phylogenetic subgroup (Hewes and Taghert, 2001). To provide new insights into the search for BRS-3 ligands, we examined whether CCHamides activate BRS-3, but we did not find any effect (data not shown).

CCHamide-1 and CCHamide-2 have been shown to be expressed predominantly in the brain and midgut (by FlyAtlas; http://www.flyatlas.org/; Chintapalli et al., 2007). In addition, CCHamide-1 and CCHamide-2 have been detected in the nervous system and midgut in a mass spectrometry study performed by Reiher et al. (2011). Therefore, CCHamides are suggested to be brain–gut peptides in insects. It is generally accepted that brain–gut peptides regulate feeding behavior in mammals (Williams et al., 2001). These peptides include neuropeptide Y, peptide YY, gastrin-releasing peptide, vasoactive intestinal peptide, adrenomedullin, cholecystokinin, galanin, glucagon-like peptide-1, and neuromedin U (Zimanyi et al., 1998; Beck, 2001). In addition, CCHamide-2 was distributed in the larval fat body (by FlyAtlas). The insect fat body is a functional counterpart of the mammalian adipose tissue and liver (Gutierrez et al., 2007). In mammal adipose tissue, leptin and adiponectin are important for feeding modulation. Therefore, we evaluated the effects of CCHamide on feeding by using the PER test in the blowfly* Phormia regina*. In flies and certain other insects, the PER test has long been used to investigate behavioral sensitivity to phagostimulative tastes (Nisimura et al., 2005). Flies extend their proboscis when the contact chemosensilla on their labella detects sweetness of sugar above a certain threshold concentration. Thus, we estimated the appetite or feeding motivation of the flies on the basis of the PER test for sucrose, in which the threshold concentration of sucrose was evaluated as an indicator of feeding sensitivity. The injection of CCHamide-2 decreased the threshold for feeding on a sucrose solution. These data suggest that CCHamide-2 stimulates the feeding motivation of flies. Indeed, administration of CCHamide-2 significantly increased the sucrose intake (Hiraguchi et al., paper in preparation). In the presence of amino acids in the diet, target-of-rapamycin complex 1 (TORC1) signaling in fat cells generates a positive messenger that is released into the hemolymph (Colombani et al., 2003). This signal reaches the brain insulin-producing cells (IPCs), where it remotely controls the secretion of *Drosophila* insulin-like peptides (Dilp). Insulin-like peptides couple growth, metabolism, longevity, and fertility with changes in nutritional availability (Gèminard et al., 2009). If CCHamide is a humoral factor that is secreted from the fat body like unpaired 2, it may play an important role in the modulation of nutrient status and growth (Rajan and Perrimon, 2012). Mice lacking functional BRS-3 develop metabolic defects and obesity (Ohki-Hamazaki et al., 1997). Therefore, the natural ligand of BRS-3 is expected to be a prominent inhibitor of appetitive behavior. The difference between CCHamide and the unknown ligand for BRS-3 with regard to feeding behavior is not clear. Further studies should de-orphanize BRS-3 by considering CCHamide by using bioinformatics or antibodies for CCHamide or *Drosophila* GPCRs.

## Conflict of Interest Statement

The authors declare that the research was conducted in the absence of any commercial or financial relationships that could be construed as a potential conflict of interest.

## Abbreviations

BRS-3: bombesin receptor subtype 3
ATRX: alpha thalassemia/mental retardation syndrome X-linked
GPCR: G protein-coupled receptors.
